# Where Are the Donors?

**Published:** 1892-10-15

**Authors:** 


### Passing Topics.

### WHERE ARE THE DONORS?

This is a question which many hospital authorities must be seriously asking, and if there be any to whose minds the inquiry appears superfluous, we must congratulate them alike upon their freedom from the anxieties and disappointments of that search so many of their fellow-workers are engaged in, and the astute manner in which they are contriving to keep all the good things to themselves.

But are there any institutions thus fortunately circumstanced? We incline to the belief that the present year, so far as it has gone, has been singularly wanting in that fertility which ensures a good harvest and renders a community contented and prosperous. Hospitals, we know, are not prosperous, and we fear that, as a natural sequence, their managers are far from contented. And that this year of all years should have failed is especially discouraging, because hospitals have never been more in evidence. Mr. Walter Besant and Mr. Egmont Hake have pleaded powerfully for them, and the Lords' Committee have pronounced an encomium upon their administration none the less pleasing because it was expected. Upon the whole, too, the Press has been kindly and sympathetic. Yet we hear lamentations upon all sides over times almost unprecedentedly bad, and some weak brethren are prepared to give up the hospital financial problem as practically insoluble.

Nevertheless hospital workers are not so far gone in despair as to clutch at every straw. They are quite capable of wishing to be saved from their friends—of a sort. Nothing but good-humoured merriment has been caused by the proposal for a "Hospital Monday," and the reasonableness of the criticism to which this suggestion has been subjected is obvious. People who do not find opportunities to minister to the hospitals upon the Sunday or the Saturday set apart for the purpose, are scarcely likely to be more complacent upon a Monday. The sentiment connected with any particular day of the week is not calculated to succeed where considerations much more powerful fail. Appeals by post are ceasing to be uniformly fruitful, and the explanation is not far to seek. The penny post has not proved an unmixed blessing, and it has provided a "fatal facility," which has been fraught with little short of persecution to the subscribing few.

It is a favourite suggestion of the teacher, that we should "try again"; and depression of spirits is occasionally averted by the cheery assurance that a good time is coming. Unhappily the hospitals are growing sick with hope deferred. Years of famine succeed one another with wearisome monotony, each leaner than that which has gone before; and the day of plenty is not yet dawning. The unfair mutterings of undiscriminating critics have something to do with this, no doubt; but after all, and notwithstanding the scorn with which, in a curiously cynical passage, the phrase is dismissed by the writer who has illuminated with a somewhat garish light the firmament of our twilight contemporary, the cause at stake is that of the "suffering poor," and while the institutions which serve it cannot hope to be safe from the vicissitudes which affect all human undertakings, it is too sacred to fail. Agricola.

